# Wolff-Parkinson-White syndrome with an unroofed coronary sinus without persistent left superior vena cava treated with catheter cryoablation

**Published:** 2008-08-01

**Authors:** Andrei Catanchin, Eduardo Castellanos, Jahangiri Marjan, David Ward

**Affiliations:** Department of Cardiovascular Sciences, St George's University of London, Cranmer Terrace, London SW17 0RE, United Kingdom

**Keywords:** Unroofed coronary sinus, WPW (Wolff-Parkinson-White) syndrome, Accessory pathway, Cryoablation, Left superior vena cava, Congenital heart defect

## Abstract

Coronary sinus anomalies are rare congenital defects which are usually coexistent with a persistent left superior vena cava and may be associated with cardiac arrhythmias. We report an unroofed coronary sinus without persistent left superior vena cava diagnosed during a catheter ablation procedure for Wolff-Parkinson-White syndrome. Diagnostic and therapeutic options and outcomes are discussed. This condition is of relevance to electrophysiologists performing catheter-based procedures, as well as cardiologists implanting coronary sinus pacing leads, who may encounter this anomaly in their practice.

## Introduction

The unroofed coronary sinus is a rare anomaly in which a section, or all, of the common wall between the coronary sinus (CS) and the left atrium is absent. Unroofed CS is usually associated with a persistent left superior vena cava (SVC) draining to the CS. During embryological development, the left anterior cardinal vein drains into the CS - failure of this vein to degenerate explains the drainage pattern of persistent left SVC into the CS. Four anatomical variants are described: completely unroofed CS with or without persistent left SVC (types I and II respectively), and partially unroofed CS in the midportion (type III) or terminal portion (type IV) [1]. Unroofed CS may be associated with other congenital heart defects [2-4]. Symptoms are related to the size of the defect and the presence or absence of a persistent left SVC, and the anatomy is well demonstrated on MRI (magnetic resonance imaging). Previous cases of CS anomalies in association with accessory pathways have been reported [5,6], however this is the first report of Wolff Parkinson White (WPW) syndrome associated with an unroofed CS without a persistent left SVC.

## Case Report

**Figure 1:**
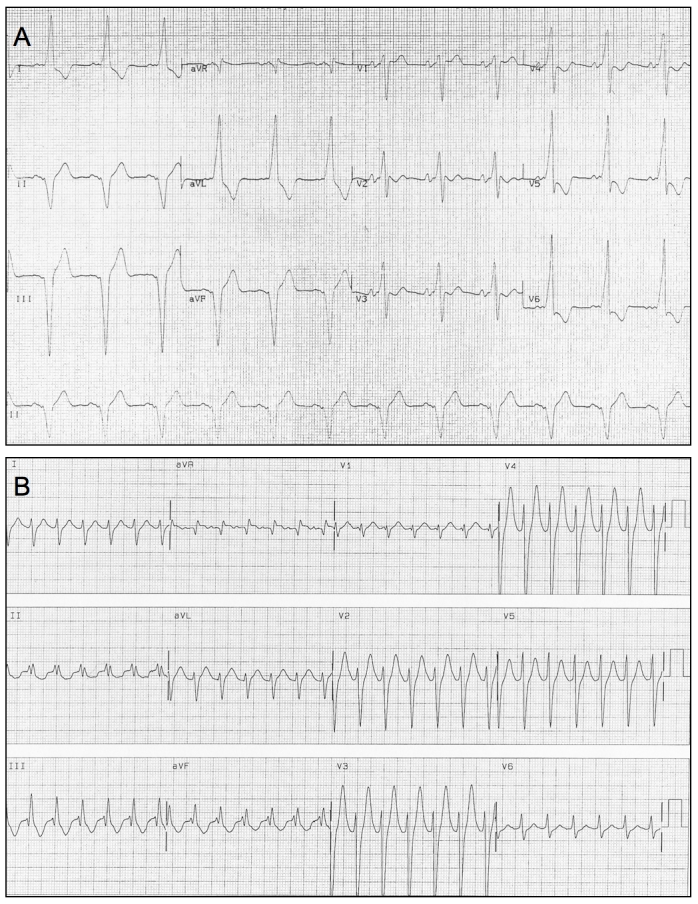
12-lead ECGs showing sinus rhythm with preexcitation (A) and orthodromic AV re-entrant tachycardia (B).

A 64 year old man was referred to our hospital with a 30 year history of palpitations. The admission 12-lead ECG in sinus rhythm demonstrated pre-excitation with a delta wave axis suggestive of a right posteroseptal accessory pathway (Figure 1).

**Figure 2:**
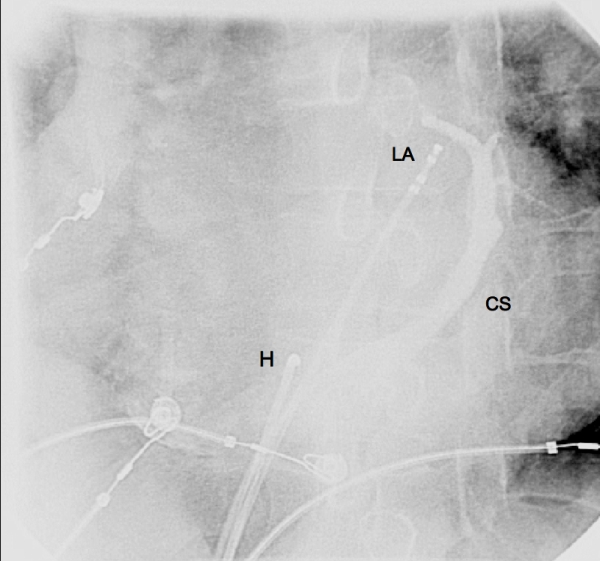
Angiography in posteroanterior projection of the coronary sinus (C) during the first electrophysiological study. A catheter is seen to reach the left atrium (LA) via a defect near the proximal CS; another is positioned in the region of the His bundle (H).

An electrophysiological study was performed. Three catheters were introduced via the right femoral vein as follows: two quadripolar 5F catheters to the right ventricular apex and coronary sinus respectively, and a quadripolar deflectable 8 mm tip Blazer ablation catheter (Boston Scientific Corp., Natick MA) was used for ablation. During catheterisation of the CS, the catheter appeared to take an unusual route and reached the left atrium; for this reason venography of the CS was performed (Figure 2).

The tricuspid annulus was mapped in sinus rhythm and earliest ventricular activation was found at the CS ostium. Equally, earliest atrial activity during orthodromic atrioventricular re-entrant tachycardia was documented at the same position. Radiofrequency energy was delivered and there was no evidence of preexcitation or accessory pathway conduction at the termination of the procedure. However, preexcitation was noted on 12-lead ECG the following day and the palpitations recurred despite treatment with flecainide.

Due to the recurrence of accessory pathway conduction and abnormal anatomic findings further investigations were undertaken. Transthoracic and transoesophageal echocardiography suggested an inter-atrial communication, possibly a primum type atrial septal defect, a large coronary sinus and right ventricular dilatation. At right heart catheterisation a left-to-right shunt was demonstrated with a shunt ratio of 2.2:1.0 and a 'step-up' in saturation from 85% in the superior vena cava to 91% in the right atrium and ventricle. MRI demonstrated a normal interatrial septum, normal tricuspid valve and inferior vena cava, no left SVC and a defect in the inferoposterior right atrium consistent with an unroofed CS. The right ventricle was dilated and a left-to-right shunt (ratio 1.8:1.0) was calculated between the left and right atria.

Given these findings the patient was referred for surgical correction. Intraoperatively, evidence of right heart volume overload and a very large coronary sinus (1.5 cm diameter) was noted. A defect was seen at the entrance of the CS into the right atrium involving the proximal CS, opening into the left atrium. No left SVC was present. The defect was repaired with an autologous pericardial patch, isolating the CS from the left atrium, and restoring normal CS drainage to the right atrium. The postoperative course was uneventful however the patient suffered recurrent palpitations despite antiarrhythmic therapy and consequently a further catheter ablation procedure was arranged.

**Figure 3:**
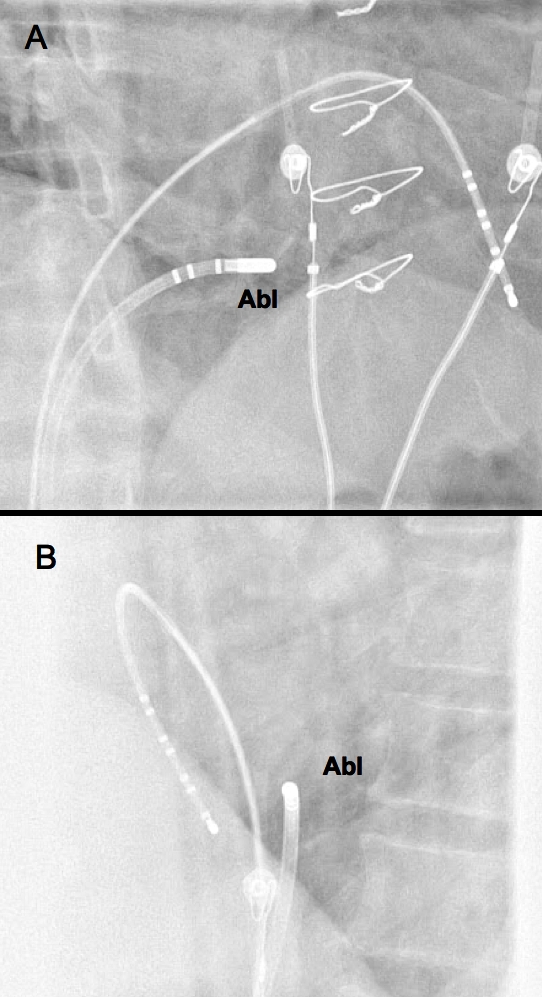
Catheter positions during successful cryoablation: ablation catheter (Abl) and right ventricular apex. Two x-ray projections are shown: right anterior oblique (A) and left anterior oblique (B)

A diagnostic octopolar catheter was advanced to the right ventricular apex and a Freezor Max cryoablation catheter (CryoCath Technologies Inc., Montreal) was used for mapping and ablation during tachycardia at a site with the shortest VA interval (Figure 3).

**Figure 4:**
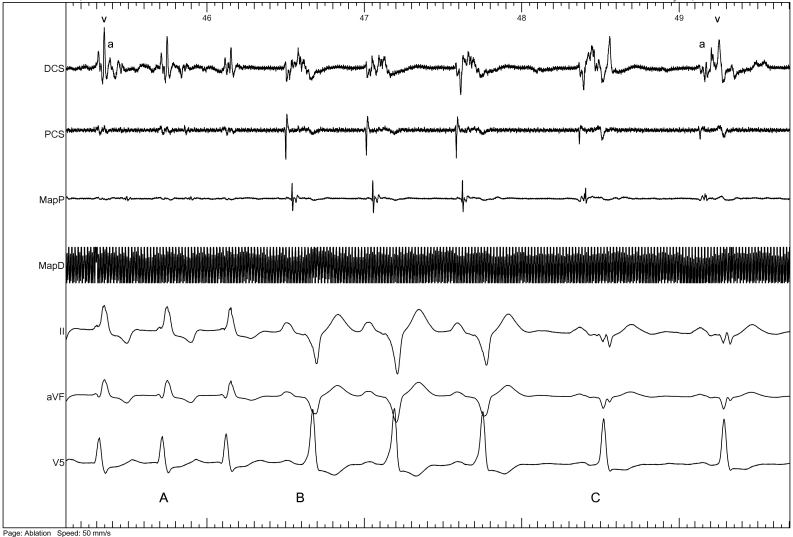
Intracardiac recordings during cryoablation showing termination of tachycardia (A), preexcited sinus rhythm (B) and finally sinus rhythm with loss of preexcitation (C). Electrograms shown are DCS (distal coronary sinus) with atrial (a) and ventricular (v) components, MapP and MapD (proximal and distal ablation catheter, respectively), and surface ECG leads II, aVF and V5.

Tachycardia terminated within 10 seconds of reaching target temperature during the first ablation delivery (-80ºC, 480 seconds), followed by sinus rhythm with preexcitation and finally loss of preexcitation (Figure 4).

Subsequent testing confirmed abolition of both antegrade and retrograde accessory pathway conduction. At 16 months' follow-up the patient was asymptomatic without evidence of preexcitation on 12-lead ECG.

## Discussion

The unroofed CS represents a rare entity in which the development of the CS is incomplete. Various authors have reported associations between coronary venous system anomalies and accessory pathways and they emphasise the importance of detailed knowledge of the venous anatomy to facilitate ablation of specific accessory pathways [5-10]. For example, Takatsuki et al report a case of atresia of the CS ostium and persistent left SVC coexistent with WPW syndrome due to a posteroseptal accessory pathway located at the blind end of the CS treated successfully with radiofrequency ablation [6] and Gaita et al report a case of WPW syndrome associated with the presence of a giant right atrial diverticulum [7]. This association between anomalies due to incomplete formation of the coronary sinus and accessory pathways may be explained by incomplete formation of the fibrous atrioventricular annulus.

There is very little reported in the literature on the association between unroofed CS and other arrhythmias. Sato et al report a case of a 2 year old girl with cor triatriatum, unroofed coronary sinus and persistent left SVC with atrial tachycardia, initially treated with catheter radiofrequency ablation and subsequently with intraoperative cryoablation, with a good outcome [8].

In the current case an unroofed CS (terminal portion, type IV) was suspected during CS catheterisation, confirmed with echocardiography and, as previously reported, demonstrated on MRI [9]. Cardiac catheterization is not necessary to make the diagnosis however in this patient it was helpful to evaluate the left-to-right shunt. Transcatheter device closure is not a feasible option, with surgical repair being the treatment of choice for symptomatic or hemodynamic indications [10-12].

Catheter cryoablation was a successful therapy for WPW in this patient, after unsuccessful radiofrequency ablation, and one perceived advantage of cryoablation over radiofrequency energy is catheter stability with attachment to the myocardium, especially if ablating during tachycardia. This is in keeping with other reports of successful cryoablation of septal accessory pathways in the presence of anatomical anomalies [13].

## Conclusion

This case represents the first report of the association between WPW syndrome and an unroofed CS without persistent left SVC. Catheter cryoablation is an effective therapeutic option, even after surgical correction of the anomaly.
